# Genome-Wide Identification, Phylogenetic and Co-Expression Analysis of *OsSET* Gene Family in Rice

**DOI:** 10.1371/journal.pone.0065426

**Published:** 2013-06-07

**Authors:** Zhanhua Lu, Xiaolong Huang, Yidan Ouyang, Jialing Yao

**Affiliations:** 1 College of Horticulture and Forestry Science, Huazhong Agricultural University, Wuhan, PR China; 2 College of Life Science and Technology, Huazhong Agricultural University, Wuhan, PR China; 3 National Key Laboratory of Crop Genetic Improvement and National Center of Plant Gene Research (Wuhan), Huazhong Agricultural University, Wuhan, PR China; University of Georgia, United States of America

## Abstract

**Background:**

SET domain is responsible for the catalytic activity of histone lysine methyltransferases (HKMTs) during developmental process. Histone lysine methylation plays a crucial and diverse regulatory function in chromatin organization and genome function. Although several *SET* genes have been identified and characterized in plants, the understanding of *OsSET* gene family in rice is still very limited.

**Methodology/Principal Findings:**

In this study, a systematic analysis was performed and revealed the presence of at least 43 *SET* genes in rice genome. Phylogenetic and structural analysis grouped SET proteins into five classes, and supposed that the domains out of SET domain were significant for the specific of histone lysine methylation, as well as the recognition of methylated histone lysine. Based on the global microarray, gene expression profile revealed that the transcripts of *OsSET* genes were accumulated differentially during vegetative and reproductive developmental stages and preferentially up or down-regulated in different tissues. *Cis*-elements identification, co-expression analysis and GO analysis of expression correlation of 12 *OsSET* genes suggested that *OsSET* genes might be involved in cell cycle regulation and feedback.

**Conclusions/Significance:**

This study will facilitate further studies on *OsSET* family and provide useful clues for functional validation of *OsSETs*.

## Introduction

SET domain, named after the three *Drosophila* proteins SUPPRESSOR OF VARIEGATION 3–9 [SU(VAR)3–9], ENHANCER OF ZESTE [E(Z)] and TRITHORAX (TRX) [1], has been known to be involved in the biochemical process of the histone lysine methyltransferases (HKMTs) [2]. It contains an approximately 130-amino acid, presenting as an evolutionarily conserved motif in chromosome proteins from yeast to mammals and higher plants [3]. It consists of two non-contiguous regions formed by N- and C-terminal ends of the primary sequence, known as SET-N and SET-C, respectively, and an insert region (SET-I) [4]. SET domain protein methyltransferases have enormous impacts on the regulation of chromatin structure and function [5], [6]. They catalyze the transfer of methyl groups from the cofactor S-adenosylmethionine (AdoMet) to specific lysine residues of protein substrates, such as the N-terminal tails of histone (H3 or H4) and the large subunit of the Rubisco holoenzyme complex [7], [8].

Baumbusch [1] first identified 39 SET domain genes in *Arabidopsis thaliana* and divided them into four classes based on the SET domains, cysteine-rich regions and additional conserved domains. Springer et al. identified 32 SET domain genes in *Arabidopsis* and 22 ones in *Zea mays*, and classified the SET domain proteins into five subfamilies, on the basis of phylogenetic analyses and domain organization [9]. It revealed that the duplication of SET domain proteins in plants was extensive and had occurred via multiple. Ng *et al.* [10] inferred that there were at least 47 SET genes in *Arabidopsis*, 35 members in maize and 34 ones in rice, based on the annotation in Pfam and ChromDB database, which were classified into seven groups. Pontvianne [11] reported that SET domain genes in *Arabidopsis* can be divided into five classes (I to V), based on their domain architectures and/or differences in enzymatic activity of SET domain-containing proteins.

Presently, a number of *SET* genes have been functionally identified in plants. Mutation of *Arabidopsis* SET domain genes resulted in phenotypic abnormalities due to the improper regulation of important developmental genes [1], [9]–[11]. *Arabidopsis CURLY LEAF* (*CLF*), a homolog of *Drosophila E(Z)* gene, was involved in the division and elongation of cells during leaf morphogenesis [12]–[14]. Further evidences revealed that CLF can directly mediate the repression of *AGAMOUS* (*AG*), *FLOWERING LOCUS C* (*FLC*) and *FLOWERING LOCUS T* (*FT*) via lysine 27 of histone H3 trimethylation (H3K27me3) and thus control floral organogenesis in a Polycomb repressive complex 2 (PRC2)[15]–[18]. *MEDEA* (*MEA*), another *E(Z)* homolog in *Arabidopsis*, is a self-controlled imprinting gene and functions in controlling the proliferation of central cell [19]–[23]. Su(VAR)3–9 homolog (SUVH) proteins KRYPTONITE (KYP, also known as SUVH4), SUVH5 and SUVH6 are shown to function in a locus specific manner to undergo H3K9 methylation and cytosine metyltransferase 3 (CMT3)-mediated non-CG DNA methylation [24]–[27]. In contrast to the E(Z) and SUVH proteins suppressing transcription, ATX1 functions as an activator of homeotic genes by lysine 4 of histone H3 (H3K4) methylation [28]–[30]. These results uncovered the extensive functions of SET proteins in plant development.

Rice is one of the major staple foods and an ideal model species of monocotyledons for functional genomics analysis. In previous studies, several SET proteins have been characterized in rice. *OsCLF* and *OsiEZ1*, both of which are *E(Z)* homologies, is expressed preferentially in young seedlings and during reproductive development [31]–[33]. SET Domain Group 714 (SDG714) and SDG728, encoding H3K9me2 histone methyltransferase, display specific functions in chromatin modification and retrotransposon repression [34], [35]. The histone methyltransferase SDG724 mediates H3K36me2/3 deposition at *MADS50* and *RFT1* and promotes flowering in rice [36]. Therefore, it is necessary to carry out a comprehensive functional exploration for *OsSET* gene family in rice.

In this study, the members of SET family in rice have been analyzed based on complete genome and protein sequences and annotations. Expression patterns and co-expression analyses were performed to discover the potential functions of *OsSET* gene family. Promoter *cis*-elements identification and the combined analysis of expression correlation suggest that most of *OsSET* genes may be cell cycle modulated and linked to the cell cycle progression by histone modifications. Our results will provide a useful reference for further functional analysis of members of *OsSET* gene family in rice.

## Results and Discussion

### Identification and Classification of *OsSET* Gene Family

In the previous report [10], 34 SET genes in rice genome were inferred, on the basis of annotation in Pfam and ChromDB database. However, based on the update Pfam and ChromDB database and MSU data, 43 SET family genes in rice were identified in our study. These 43 *OsSET* genes were named from *OsSET1* to *OsSET43* according to their positions on chromosomes. Nine genes, *OsSET12, OsSET13, OsSET18, OsSET23, OsSET29, OsSET31, OsSET36, OsSET37* and *OsSET39*, were novel *OsSET* genes compared to the ones in previous study. *OsSET* genes vary substantially in the size of their encoded proteins and their physicochemical properties (Table S1). *OsSET33* has 25 exons and 24 introns, whereas *OsSET14*, *OsSET20* and *OsSET30* have no intron. The position of the SET domain also varies within the proteins. The shortest OsSET protein is OsSET9 with 231 amino acids, while the longest one is OsSET30 with 1292 amino acids. EXPASY analysis showed a large variation in isoelectric point (pI) values (ranging from 4.4119 to 9.2625) and molecular weights (ranging from 110.892 kDa to 138.5423 kDa). Nevertheless, only 3 (*OsSET4, OsSET14* and *OsSET29*) of the 43 *OsSET* genes were predicted to be stable proteins. Details on other parameters of protein sequences were shown in Table S1.

### Chromosomal Localization and Gene Duplication

**Figure 1:**
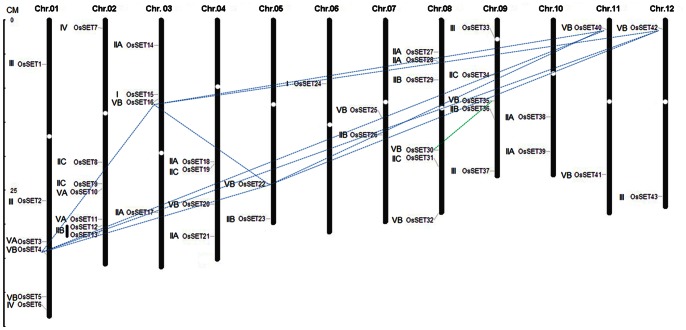
Chromosomal distribution, and tandem and segmental genome duplications of the *OsSET* gene family. The scale on the left is in megabases (Mb). The ovals on the chromosomes (vertical bars) indicate the positions of centromeres; the chromosome numbers are shown on the top of each bar. The segmental duplication genes are connected by a dotted line.

The genomic distribution of *OsSET* genes was determined by their chromosomal positions on rice chromosome. Totally, 43 *OsSET* genes were dispersed on the 12 chromosomes, presenting unevenly in all regions of the chromosomes. A brief diagrammatic representation of chromosomal distribution of *OsSET* genes was described (**Figure 1**, the exact position on rice chromosome pseudomolecules was given in Table S1). Seven *OsSET* genes are on chromosome 2, six *OsSET* genes are on chromosome 1 and 8, respectively; five *OsSET* genes are on chromosome 9; four *OsSET* genes are on chromosome 3 and chromosome 4; two *OsSET* genes are on chromosome 5, 7, 10, 11, 12, respectively; only one *OsSET* gene is on chromosome 6.

During the evolution of a gene family, segmental duplication and tandem duplication play important roles in generating new members [37]. Therefore, both segmental and tandem duplication events were investigated for elucidating the potential mechanism of evolution of *OsSET* gene family. Analysis of the MSU RGAP rice segmental duplication database revealed that 10 pairs of *OsSET* genes could be assigned to MSU RGAP segmental duplication blocks. The overall similarity of the cDNA sequences of these genes ranged from 25.6% to 77.0% and all of them were found to have their counterparts on duplicated segments (Table S2). Interestingly, these duplicated segments can be clustered in 2 groups. Five *OsSET* genes (*OsSET4*, *OsSET16*, *OsSET22*, *OsSET40* and *OsSET42*), which had high overall identities between each other, belonged to the same group. While the overall identity between *OsSET30* and *OsSET35* was 52.0%, and was included in another group. None of the *OsSET* genes seemed to be generated from tandem duplications in our analysis. These results implicated that much of the diversity of the *OsSET* gene family in rice was mainly due to the segmental duplication events.

### Phylogenetic and Structural Analysis of *OsSET* Gene Family

**Figure 2:**
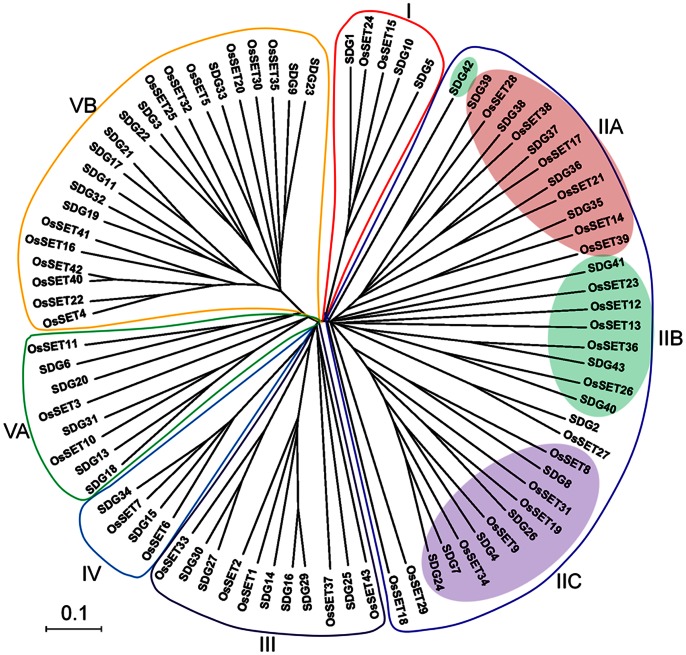
Phylogenetic analysis of *Arabidopsis* and rice SET proteins. Phylogenetic tree of rice and *Arabidopsis* SET proteins. An unrooted NJ tree of rice and *Arabidopsis* SET proteins is shown. The six classes are marked by different colors. Scale bar represents 0.1 amino acid substitution per site.

To determine the evolutionary relationships of *SET* family genes between rice and *Arabidopsis*, an unrooted phylogenetic tree was constructed from alignments of their full-length protein sequences. The latest data showed that *Arabidopsis* SET family genes can be divided into five classes (I to V), based on their domain architectures and/or differences in enzymatic activity of SET domain-containing proteins [11]. Coincidently, our phylogenetic analysis and their domain architectures support the classification of rice and *Arabidopsis* SET gene family into five classes (**Figure 2** **,** **3**; Table S1).

**Figure 3:**
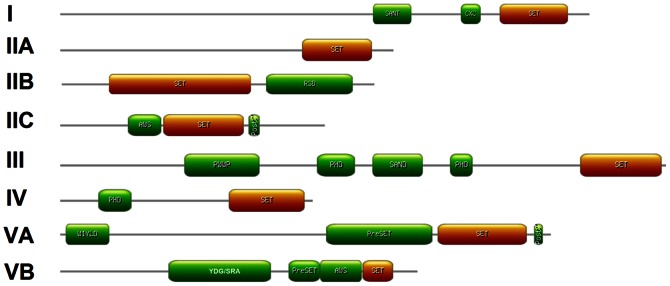
Structure of representative OsSET proteins from each subfamily. The protein structure is based on the presence of OsSET and other additional domains as identified by Pfam. Subfamily name of each corresponding protein belonged to are given on the left. The length and order of domains represent actual situation in each protein.

Class I SET proteins include 2 rice OsSET proteins and 3 *Arabidopsis* SET proteins. OsSET15/OsiEZ1 and OsSET24/OsCLF are the homologs of *Arabidopsis* SWINGER (SWN)/SDG10 and CLF/SDG1, respectively. No *Arabidopsis* MEDEA (MEA)/SDG5 homolog was found in rice. In addition to the C-terminal SET domain, SANT domain (Swi3, Ada2, N-Cor, and TFIIIB DNA binding domain) and cysteine rich CXC domain were found in this subfamily. This result is in agreement with previous studies [10]. Recent studies suggested that SANT domains might be a histone-tail-binding module [38], [39]. It is reported that E(Z)-like proteins are components of PRC2 complexes and function as transcriptional repressors by H3K27me3 in diverse eukaryotes [13], [16], [21], [22]. Therefore, OsSET15/OsiEZ1 and OsSET24/OsCLF may have H3K27me3 activities by these conserved domains.

Class II subfamily can be divided into three clusters of IIA, B and C based on their domains, which is also in accordant with the previous reports [9], [10]. Eight members in rice (OsSET14, OsSET17, OsSET18, OsSET21, OsSET27, OsSET28, OsSET38 and OsSET39) and five in *Arabidopsis* (SDG35–39) belonged to Class IIA, which only contain the SET domain. Class IIB proteins (OsSET12, OsSET13, OsSET23, OsSET26, OsSET36, SDG40, SDG41, SDG42, SDG43) have a Rubisco LSMT substrate-binding domain (RSB domain), which allows the binding of the protein to its substrate, such as the N-terminal tails of histones H3 and H4 and the large subunit of the Rubisco holoenzyme complex [7]. Class IIC has five SET proteins in rice (OsSET8, OsSET9, OsSET19, OsSET31 and OsSET34/SDG724) and five in *Arabidopsis* (ABSENT, SMALL, OR HOMEOTIC DISCS 1 HOMOLOG 1 (ASHH1)/SDG26, ASHH2/SDG8, ASHH3/SDG7, ASHH4/SDG24 and ASH1-related 3 (ASHR3)/SDG4 [40]–[42],). All proteins except OsSET31 in class IIC have an AWS domain **(A**ssociated **W**ith **S**ET, a sub unit of pre-SET domain) [10]. Five of the members in class IIC have an additional cysteine-rich post-SET domain. Although some of the class II subfamily genes have been demonstrated to methylate H3K36 at the region of actively transcribed genes [43], the functions of the additional domains are still little known.

Class III HKMTs consist of four rice SET members (OsSET1, OsSET2, OsSET33 and OsSET37) and seven *Arabidopsis* genes. Five of the *Arabidopsis* genes encode homologs of Trithorax (trxG), which named as *Arabidopsis* Trithorax-like protein (ATX1/SDG27, ATX2/SDG30, ATX3/SDG14, ATX4/SDG16 and ATX5/SDG29), while another two genes, ATXR3/SDG2 and ATXR7/SDG25, are ATX-RELATED (ATXR) genes [44]. Class III subfamily genes have several additional highly conserved protein domains, including PWWP, FYRN/C and plant homeodomain (PHD). The PWWP domain was shown to be a DNA or methyl-lysine histone binding domain [45]–[47]. In *Arabidopsis*, class III proteins are able to methylate H3K4me2/3, acting as antagonistic regulators with Polycomb Group (PcG) proteins to maintain transcriptional OFF and ON states of their target genes [48], [49]. Interestingly, PHD finger is considered to be specific and highly robust binding modules for H3K4me2/3 in humans and plants, resulting the recruitment of basal transcriptional active factor(s)[50]–[53]. ATX1 has been demonstrated to interact with ASHH1/SDG26, suggesting that trxG complexes could involve different sets of histone lysine methyltransferases in *Arabidopsis* [40]. These results provide an efficient insight for functional identification of trxG in rice.

OsSET6, OsSET7, ATXR5/SDG15 and ATXR6/SDG34 are included in class IV, which characterized with a PHD domain and a SET domain. ATXR5 and ATXR6 are involved in DNA replication [54]. Although class IV proteins are near to class III on their evolutionary relationship, they are distinct from class III for the absence of PWWP domain. The result suggests that the PWWP domain may be crucial for the antagonistic mechanism between PcG and trxG.

Class V proteins are characterized by the presence of pre-SET and SET domains. This class can be divided into VA [SU(VAR)3–9 (SUVR)] and VB (SUVH) for the latter having an YDG/SRA domain [1]. Class VA contains three OsSET proteins (OsSET3, OsSET10 and OsSET11) and 5 SU(VAR)3–9 (SUVR) proteins (SUVR1/SDG13, SUVR2/SDG18, SUVR3/SDG20, SUVR4/SDG31 and SUVR5/SDG6). OsSET10, SDG18 and SDG31 also have an N-teminal plant-specific domain, WIYLD, which binds ubiquitin and enables conversion of H3K9me1 to H3K9me3 [55]. Subfamily VB consists of 12 OsSET proteins (OsSET5/SDG714, OsSET14, OsSET16, OsSET20, OsSET22/SDG728, OsSET25, OsSET30/SDG710, OsSET32, OsSET35/SDG727, OsSET40, OsSET41, OsSET42) and 9 SUVH proteins (SUVH1/SDG32, SUVH2/SDG3, SUVH3/SDG19, SUVH4/KYP/SDG33, SUVH5/SDG9, SUVH6/SDG23, SUVH7/SDG17, SUVH8/SDG21, SUVH9/SDG22 and SDG11). It has reported that the YDG/SRA domain can mediate epigenetic inheritance by recruiting histone deacetylase (HDAC), DNA methyltransferase (DNMT) to methyl-CpG site [56]–[58]. Similarly, the YDG/SRA domain of KYP, SUVH5 and SUVH6 binds directly to methylated DNA at both CpG and non-CpG site, thereafter, providing a binding site for CMT3 via its chromodomain to CHG methylation [59]. OsSET5/SDG714, an H3K9 methyltransferase, is also involved in DNA methylation in rice [35], [60], which implies a similar mechanism between class VB OsSET proteins and DNA methylation. SUVR5 establishes the heterochromatic state by H3K9me2 deposition in a DNA methylation–independent manner through zinc fingers [61]. However, no such DNA binding domain was identified in rice SUVR like proteins. Therefore, there must be a distinct mechanism for SUVR in rice.

**Figure 4:**
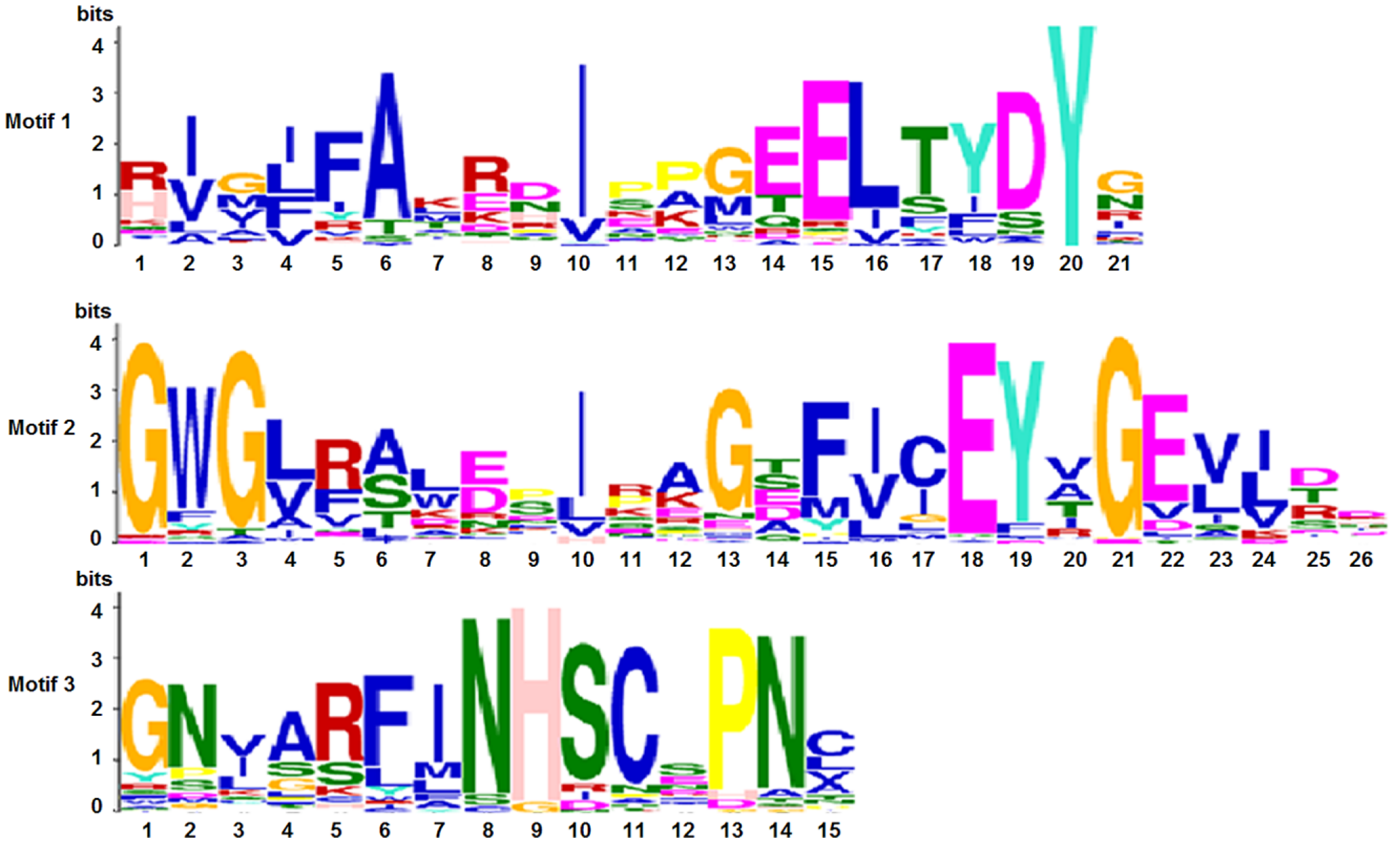
Sequence LOGOs for each motif of SET domains using the MEME algorithm. MEME motifs are displayed by stacks of letters at each position. The total height of the stack is the “information content” of that position in the motif in bits. The height of the individual letters in a stack is the probability of the letter at that position multiplied by the total information content of the stack. X- and Y-axis represents the width of motif and the bits of each letters, respectively.

Because SET domain is essential for the catalytic activity of SET proteins, the MEME motif search tool was employed to identify the conserved motifs of SET domains from 84 SET proteins in rice and *Arabidopsis*. Three distinct motifs, motifs 2, 3 and 1, were located orderly at SET-N, SET-I and SET-C region of SET domain, respectively (**Figure 4**). 55 out of 84 (65.5%) SET proteins have motifs 1, 2 and 3. 17 (20.2%) SET proteins only have motifs 1 and 3. OsSET29 and OsSET39 have motifs 2 and 3. OsSET12, OsSET13 and OsSET23 only have motif 1. OsSET3 and OsSET16 only have motif 2. The other five SET proteins (OsSET26, OsSET36, SDG40, SDG42 and SDG43) have none of the three motifs. Interestingly, 24 out of 29 (82.8%) proteins that have no more than 2 motifs are subfamily IIA or IIB members, which showed diversity in these two subfamilies.

### Identification of *Cis*-elements in *OsSET* Gene Promoters

To understand the molecular mechanism of *OsSET* genes in transcriptional regulation, *cis*-elememts at the promoter regions were identified (Table S3). As a result, 255 *cis*-elements were obtained. In addition to basic TATA-box and CCAAT box, *cis*-elements such as MYB recognition, auxin responsive, gibberellin (GA) response, abscisic acid (ABA) responsive and E2F-binding site were found at the promoter regions of *OsSET* genes. It is well known that E2F transcription factors control the cell cycle by regulating transcription of genes required for DNA replication and cell cycle [62]. Many investigations show that the E2F targets have one or more consensus DNA sequence of E2F binding sites [63]–[65]. It was reported that *ATX1/SDG27*, *ATXR5/SDG15*, and *ATXR6/SDG34* in *Arabidopsis*, *OsSET6,* and *OsSET7* in rice, and lots of other cell cycle or DNA replication related genes were considered as E2F targets for their E2F binding *cis*-elements [54], [63], [64]. While, in our analysis, we found 32 *OsSET* genes had E2F binding site (E2FCONSENSUS, SITEIIBOSPCNA, SITEIOSPCNA, E2FANTRNR, E2F1OSPCNA, E2FAT, PE2FNTRNR1A) (Table. 1). Therefore, the analyses revealed that most of the *OsSET* genes might be regulated by E2F transcription factors.

**Table 1:** Conserved E2F binding *cis*-elements analysis of the *OsSET* gene promoters.

Accession Number^a^	ID^b^	Sequences
S000217	SITEIIBOSPCNA	TGGTCCCAC
S000224	SITEIOSPCNA	CCAGGTGG
S000366	E2FANTRNR	TTTCCCGC
S000396	E2F1OSPCNA	GCGGGAAA
S000417	E2FAT	TYTCCCGCC; Y = T/C
S000455	PE2FNTRNR1A	ATTCGCGC
S000476	E2FCONSENSUS	WTTSSCSS; W = A/T; S = C/G

a Accession number of *cis*-element in PLACE database.

b Locus identity number of *cis*-element in PLACE database.

### Expression Profiling of *OsSET* Genes in Rice

**Figure 5:**
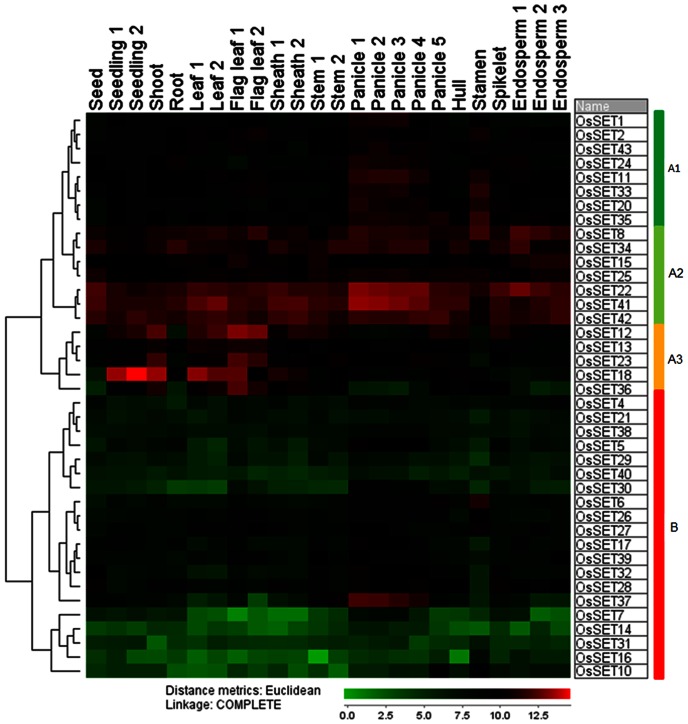
Expression patterns of *OsSET* genes during the life cycle of the rice plant. Hierarchical cluster displays the expression profile for 40 *OsSET* genes with matching probesets in the Affymetrix microarray. (Color bar at the base represents log2 expression values: green, representing low expression; black, medium expression; red, high expression).

To investigate the transcript accumulation of *OsSET* genes in the entire life cycle, the expression profiling covering 24 developmental stages (Table S4) in Minghui 63 were analyzed using Affymetrix rice microarray data from CREP database. A hierarchical cluster displaying the logarithm of average signal values for the 40 *OsSET* genes were generated. Distinctly, the expression patterns of *OsSET* genes could be classified into two major groups (**Figure 5**). 20 genes belonged to Group I, most of which showed high transcript accumulations (average expression signal from 777.3 to 4211.1) in the tissues analyzed. *OsSET41* had the highest expression level in the entire life cycle. These genes could be further divided into three subgroups, subgroup A1–3. Subgroup A1 consists of 8 genes, which have high expression level in panicles and/or stamen. Subgroup A2 has 7 *OsSET* genes, all of which show relative high expression level in almost all tissues analyzed. Subgroup A3 has 5 *OsSET* genes, which display higher expression in vegetative tissues than in reproductive tissues. Group B contains 20 genes, exhibiting relative low expression signals in most tissues or preferential expressions in some tissues. *OsSET5*, *OsSET6*, *OsSET10*, *OsSET17*, *OsSET27*, *OsSET28*, *OsSET32* and *OsSET37* showed high expression in panicles; *OsSET6*, *OsSET10* and *OsSET27* expressed relatively higher in stamen, In addition, *OsSET28* in spikelet and seed, *OsSET32* in seed, *OsSET37* in stem showed tissue-specific expressions.

**Figure 6:**
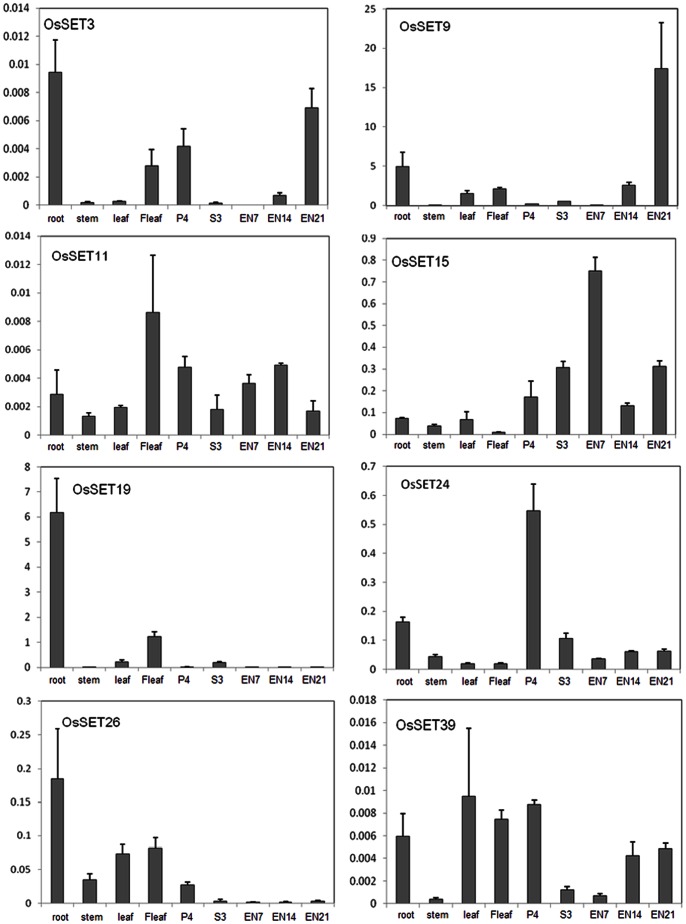
Relative expression of eight *OsSET* genes in Zhonghua11. root, roots at trefoil stage; stem, stems at heading stage; leaf, leafs at at trefoil stage; Fleaf, flag leafs at heading stage; P4, panicles at meiosis stage of young panicle development; S3, seeds of 3 days after pollination; EN, endosperm, the number followed it means the days after pollination.

The expression patterns of some *OsSET* genes were further confirmed by real-time PCR analysis. The expression levels of *OsSET3*, *OsSET9* and *OsSET19*, which have no probeset information in CREP database, were detected in different tissues (**Figure 6**). *OsSET3* shows a relative high expression in root, flag leafs, panicles and mature endosperm. Both *OsSET9* and *OsSET19* are included in class II, however, their expression patterns are divergent: *OsSET9* are highly expressed in the later stage of endosperm (21 days after pollination), whereas the expression of *OsSET19* enriched in young leaf and decreased in endosperm. The expression levels of *OsSET11*, *OsSET24/OsCLF* and *OsSET15*/*OsiEZ1* are also detected, which are in accordance with the microarray data. *OsSET26* has a low expression pattern in microarray analysis, whereas our quantitative PCR result shows a relative higher expression level in vegetative stage than reproductive stage. *OsSET39* expresses relatively higher in root, leaf and panicles, and the expression is enriched in the developing endosperm, implying multiple functions in plants development.

**Figure 7:**
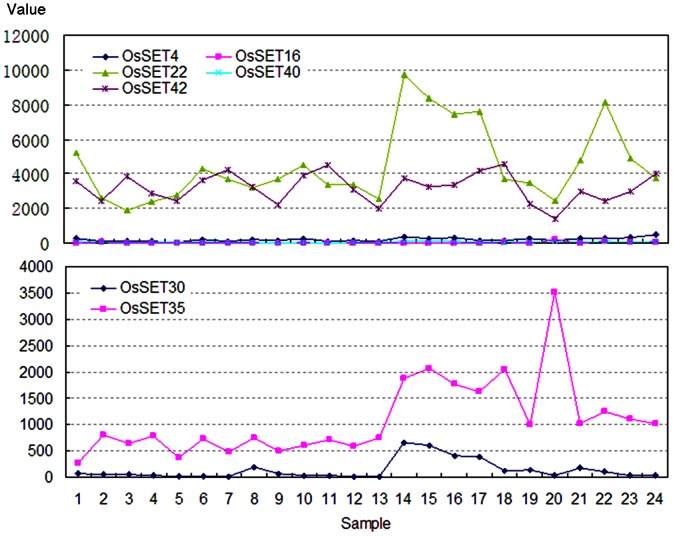
Expression patterns of *OsSET* genes found in segmentally duplicated regions of the rice genome. X-axis represents the developmental stages as given in the following table. Y-axis represents the raw expression values obtained from microarray.

On the purpose of revealing more information in *OsSET* gene expression pattern, genes that showed differential expression during various developmental stages in comparison to seed were analyzed. Genes that considered as preferential expression in a given stage showed tremendous differences (Fig 7, Table S5). Up-regulated genes mainly accumulated in panicles and stamen, suggesting that *OsSET* genes may participate in various molecular pathways in flowering development. Surprisingly, although down-regulated genes accumulated in seedlings, they were activated in stamen, either. These microarray and real-time PCR results indicate that *OsSET* genes may play essential roles through the life cycle of rice.

**Figure 8:**
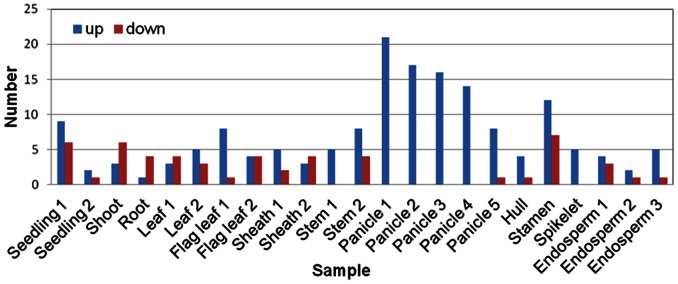
Differential expressions of *OsSET* genes in different stages in Minghui 63 based on microarray analysis. Differential expression genes have been taken p value less than 0.05 and fold change >2 or <0.5. When fold change >2, regulation is up, and when fold change <0.5, regulation is down.

The expression patterns of segmentally duplicated *OsSET* genes were examined by Affymetrix microarray data. Probesets were available for all segmental duplication genes in microarray data. A comparison of expression level revealed that a pair of segmental duplicated genes always showed similar expression pattern, although one of the copy showed low expression level, or was not expressed at significant levels in most of the tissues (**Figure 8**). In the group of *OsSET4*, *OsSET16*, *OsSET22*, *OsSET40* and *OsSET42* (**Figure 8A**), for instance, 2 out of 5 genes had a similarly high expression level. The resemblance also existed in the *OsSET30* and *OsSET35* group (**Figure 8B**). We might therefore infer that immediately after segmental duplication, the two copies of genes might be functionally redundant. However, only one of them is functional retained while the other degenerates into a pseudogene eventually.

### Responses of *OsSET* genes under NAA, KT, and GA3 Treatments

Phytohormones play critical roles in plant growth and development. To investigate the *OsSET* genes in response to phytohormone treatment, differential expression analysis was performed. As a result, 9 *OsSET* genes that were differentially expressed under one or more of the phytohormone NAA, KT, GA3 in seedlings in trefoil stage, compared with the control without treatment. The fold change values with respect to control were given in Table S4. Three *OsSET* genes showed differential expression under all three phytohormone treatments, among which *OsSET23* and *OsSET36* were up-regulated, whereas *OsSET18* was down-regulated. *OsSET2* and *OsSET16* were up-regulated to KT, and *OsSET24* and *OsSET34* were up-regulated to GA3 treatment. In contrast, *OsSET17* and *OsSET37* were down-regulated specifically to KT and GA3 treatment, respectively. The expression profile of the remaining genes in response to NAA, KT, and GA3 was not significant. These results partially in accordance with the *cis*-elements analysis, showing that the above 9 *OsSET* genes have one or more GA responsive elements (Table. S3).

### Identification and Functional Annotation of Genes Co-expressed with *OsSET* Genes

Co-expression analysis has been successfully exploited to identify functional transcription regulators in *Arabidopsis*, rice and other organisms [66], [67]. Hence, in order to disinter more information of the *OsSET* genes, 40 *OsSET* genes with matching probeset were selected as “guide genes” to identify the co-expressed genes using expression data from CREP database, with an absolute value of the Pearson correlation coefficient (PCC) greater than 0.75 (α = 0.05) [68], [69]. As a result, 2390 genes whose expression pattern tightly correlated with 30 *OsSET* members were extracted (Table S6a).

**Figure 9:**
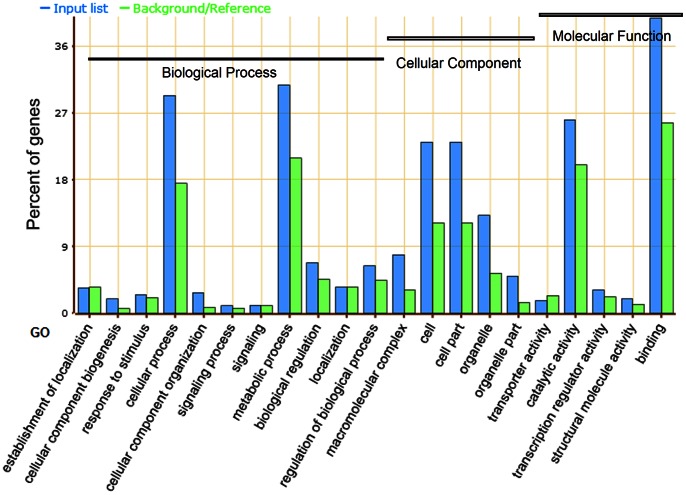
Enriched GO analysis of genes that co-expressed with *OsSETs*. X-axis represents the GO annotation. Y-axis represents the percentage of GO annotation. Three categories of GO annotation are biological process, cellular component and molecular function. Input list represents genes analyzed, and the references/background represents all genes in agriGO database.

We next analyzed the GO annotations assigned to these genes by agriGO tools. The enriched GO annotations particularly concentrate on cellular process, cellular component biogenesis and organization, biological regulation and metabolic process (**Figure 9**, Table S6b). They encode proteins as macromolecular complex (protein complex, DNA polymerase, protein-DNA complex) in cell or organelle, substantially. The molecular functions tightly associated with them are catalytic activity, transcription regulator and binding. These results suggest that the functions of *OsSET* genes may be associated with DNA replication and gene transcription.

As is generally known, SET domain proteins have comprehensive impacts on the regulation of chromatin structure and function [5], [6]. Therefore, we focused on 503 out of 2390 co-expression genes which may be associated with epigenetic regulation (histone genes, cell cycle related genes, DNA replication, transcription factor, chromatin reassemble related genes and so on. Table S6c). After the recalculation and student-*t* test of PCC, 450 genes were co-expressed with 29 *OsSET* genes. In addition, 13 *OsSET* genes were co-expressed with each other tightly. Except for *OsSET28*, the other 12 *OsSET* genes have close expression relationships with histone genes, cell cycle control related genes and chromatin assemble factors, anther-specific proline-rich protein (*APG*) genes, DNA replication related genes and so on. The result gives informative clues in functional characterization of these *OsSET* genes.

### OsSET Proteins May Be Involved in Cell Cycle Regulating by Histone Modification

**Figure 10:**
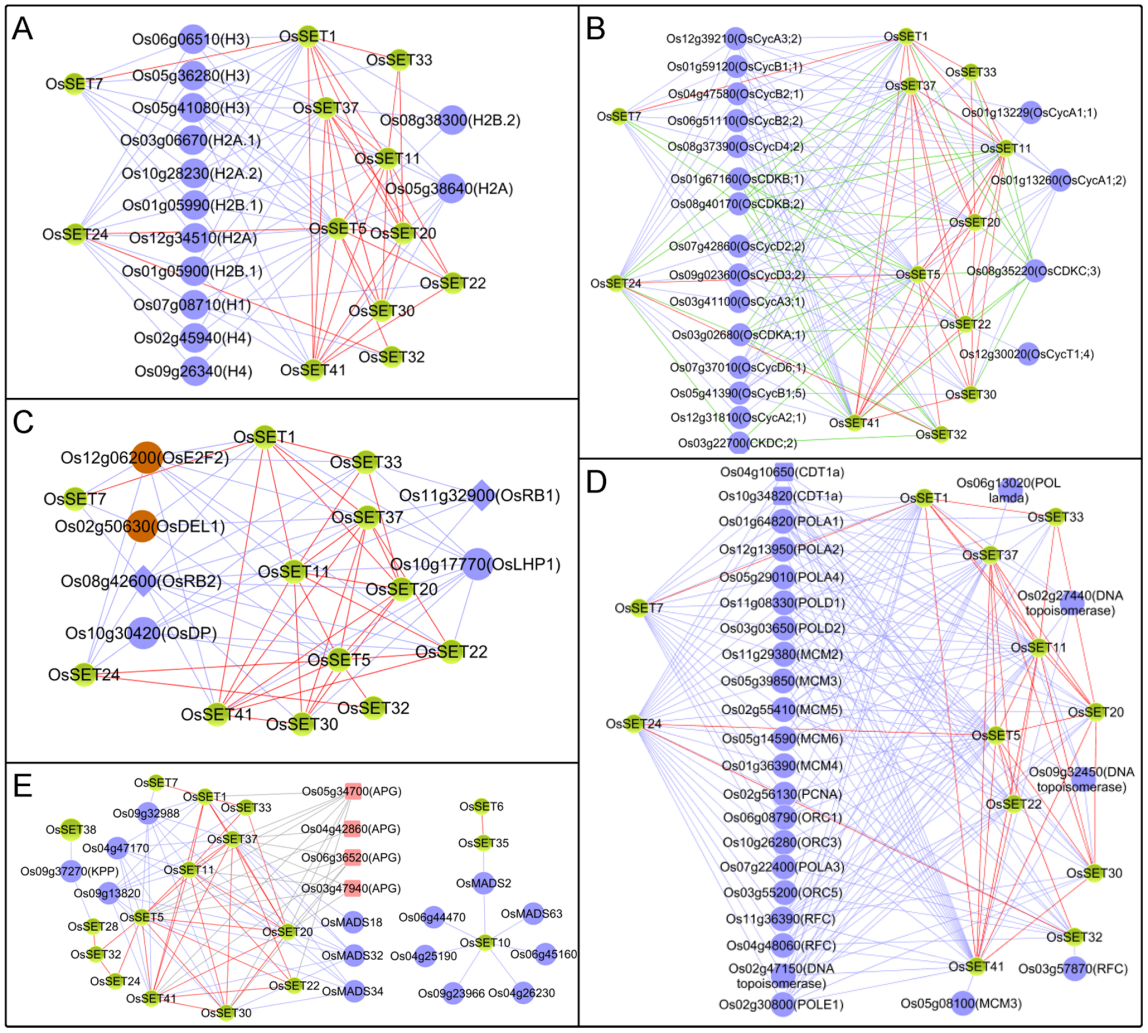
Co-expression network of *OsSET* genes. A. The co-expression relationship among *OsSET* genes and histone genes (H2A, H2B.1, H2B.2, H3 and H4). B. Network of *OsSET* genes and cell division related genes. C. Co-expression network of *OsSET* and RB-E2F/DB pathway genes. D. A co-expression network regarding the *OsSET* genes and DNA replication factors. E. A co-expression network concerning *OsSET* genes and genes involved in flower development.

The visualized figure in **Figure 10A** shows the close relationships between 12 *OsSET* genes and 13 histone protein genes (containing H1, H2A, H2B.1, H2B.2, H3 and H4). In this network, *OsSET5* is co-expressed with all of these histone genes, *OsSET24* is co-expressed with 11 histone genes, *OsSET37* has 9 co-expressed histone genes, *OsSET7* has 7 ones, both *OsSET11* and *OsSET44* have 5 ones, while others has 1 or 2 co-expressed histone genes. *OsSET5* and other five genes (*OsSET20*, *OsSET22*, *OsSET30*, *OsSET32* and *OsSET41*) belong to class VB. *OsSET24/OsCLF* is a class I member. *OsSET37* and other two genes (*OsSET1*, *OsSET33*) belong to class III. *OsSET7* is a class IV gene. *OsSET11* is a class VA gene. This co-expression network indicated that OsSET protein may not only modify histone lysine, but also be involved in multiple histone site modification directly or indirectly. Because histone proteins are essential for the packaging of newly synthesized DNA into chromosomes [70], we speculated that OsSET proteins may be relevant to cell cycle regulation.

Subsequently, we found that the 12 *OsSET* genes in **Figure 9A** were co-expressed with 19 cell cycle related genes, simultaneously (**Figure 10B**). These cell cycle related genes include 14 cyclin genes (*Cycs*, A-, B- and D- Type), and 5 cyclin-dependent kinase genes (CDKs, A-, B- and C-Type). It is noted that *OsSET5*, *OsSET24* and *OsSET37*, which are co-expressed with most of histone genes, are also tightly co-expressed with various kinds of *Cycs* and *CDKs*. While *OsSET22* and *OsSET32* are co-expressed with A- and D-type Cycs, the *OsSET33* is only co-expressed with D-type Cycs (*OsCycD2;2* and *OsCycD3;2*). *OsSET20* and *OsSET33* are co-expressed with *OsCDKB3;2* and *OsCDKC;3*. *OsSET7* is co-expressed with *OsCDKB;1* and *OsCDKB;2*. *OsSET1* and *OsSET32* are co-expressed with *CDKC;3*.

Usually, Cycs and CDKs act as complex at a precise time and drive the cell cycle progression by phosphorylating downstream target proteins. Cell cycle progression is critical for the maintenance of epigenetic marks and for allowing the daughter products to acquire a distinct epigenetic landscape [71]. Thus, the relevance might exist between SET, histone and cell cycle related protein. In human, it has been confirmed that Retinoblastoma (RB) can target H3K9 methylation to cyclin E promoter by SET-domain protein, Suv39H1, resulting in heterochromatin protein 1 (HP1) binding and silencing [72]. In higher eukaryotes, cell cycle is mainly controlled by E2F transcription factors, which acts through a conserved RB-E2F/DP pathway (DP, related to the E2F family that can dimerize with E2F members)[64], [73]–[75].

In *Arabidopsis*, CDKAs/CycDs complex can activate the expression of E2F/DP targets by phosphorylating RB and further releasing RB from its cooperator E2F/DP complex [76]–[78]. Genome-wide identification and expression analysis have found conserved *E2F*, *RB*, *Cycs* and *CDKs* in rice [79]–[81]. In our performances, two homologies of *E2F* (*OsDEL1* and *OsE2F2*) and two *RB* homologies (*OsRB1* and *OsRB2*) were found to be co-expressed with these 12 *OsSET* genes (**Figure 10C**). In consequence, these 12 OsSET proteins may be required for the RB-E2F/DP pathway during cell cycle progression. Although there is no direct evidence showing the connection between SET proteins and RB *in vivo* in plant, the CLF of *Arabidopsis* can bind the RB proteins both in maize and human [82], Shen et al proposed that HP1-HKMT-RB-E2F/DP complexes could repress E2F targets in plants [73]. In co-expression network of this study, the rice PcG gene *OsSET24/OsCLF*, a homology of *Arabidopsis CLF*, is co-expresses with *OsE2F2*, *OsDEL1*, *OsRB2* and *OsDP* tightly. Thus, it is possible that a similar RB-PRC2 complex functions in the cell cycle regulation of rice. *OsSETs* are co-expressed with histone and cell cycle related genes simultaneously, implying that more HKMTs may be associated with the regulation of cyclins via histone modifications. Therefore, our co-expression results might provide light in the relationship between these OsSET proteins and RB-E2F/DB complex**.**

### *OsSETs* May Function in Reproductive Development of Rice

Floral organ identity in plants are controlled by combinations of activities mediated by MADS box genes, some of which were identified in our co-expression analysis (**Figure 10E**). *OsMADS18* is co-expressed with *OsSET20* and *OsSET37. OsMADS32* is co-expressed with *OsSET1, OsSET5, OsSET11, OsSET20, OsSET30* and *OsSET41*. *OsMADS34* is a member in *SEPALLATA (SEP)* subfamily [83], [84], which is co-expressed with *OsSET5*, *OsSET11*, *OsSET20*, *OsSET30* and *OsSET37*. Recent research revealed that *OsMADS18*, one of *APETALA1 (AP1)/FRUITFULL (FUL)-*like genes, was induced in the shoot apical meristem **(**SAM) during meristem phase transition, which acted co-ordinately in the meristem to specify the identity of the inflorescence meristem (IM) downstream of the florigen signal [85]. The expression domains of *OsMADS32* are mainly restricted to the marginal region of the palea and inner floral organs, showing its contribution on floral organ identity in rice [**86**]. *OsMADS34* plays a role in the early development of spikelet formation [84]. In the above co-expression network, *OsSET20* is co-expressed with three MADS box genes, while *OsSET1* is co-expressed with one MADS box gene. Besides, another five *SET* genes, *OsSET5*, *OsSET11*, *OsSET20*, *OsSET30* and *OsSET37*, are co-expressed with two MADS box genes. Therefore, it is possible that these *OsSETs* may take part in flowering transition and early floral development in rice.

In the co-expression network (**Figure 10E**), 8 *OsSET* genes are correlated with 4 APG-like protein genes, while *OsSET5* and *OsSET37* are co-expressed with four *APG* genes. Noticeably, the *APG* gene *Os05g34700* is co-expressed with 8 *OsSET* genes. The transcripts of these 8 *OsSET* genes accumulate in young panicles. It was reported that *APG* transcript was confined to anther during microspore development in *Brassica. napus* flower buds [87]. It was also suggested that five *APG* genes in *Silene latifolia* were related to anther fertility, which were required for development of fertile pollen [88]. Hence, we might infer that these *OsSET* genes are involved in rice reproductive development though the regulation of *OsAPGs* during microsporogenesis stage.

Except for the above 13 *OsSET* genes, the other genes were also characterized by co-expression analysis. For example, *OsSET6*, *OsSET10*, *OsSET16* and *OsSET35* have a high expression level in stamen, and their co-expression genes include transcription factor, binding protein, pollen allergen and so on (**Figure 5** and **Figure 10E**). Among them, *OsSET6*, *OsSET10* and *OsSET16* are co-expressed with a cyclin gene. Meanwhile, *OsSET10* is co-expressed with five pollen allergen genes and 2 MADS-box genes (*OsMADS2* and *OsMADS63*). *OsSET35* is co-expressed with *OsMADS2*. The previous report showed that *OsMADS2* transcript was first observed in the region where stamen primordia were formed, and then appeared in the lodicule primordia as well as the stamen primordia [89], [90]. *OsMADS63* is the homolog of *Arabidopsis AGL66*, which encodes a MIKC*-type DNA binding factor as heterodimer affecting pollen viability, germination, and pollen tube growth [91]. Our analysis suggests that these four *OsSET* members might affect on the development of male gametophyte.

### Conclusions

In conclusion, 43 *OsSET* genes can be classified into five classes as supported by phylogeny and conserved domains organization. Phylogenetic and structural analysis indicated that the domains beyond SET domain were significant for their specific functions. The expression analysis revealed that *OsSET* genes might participate in various molecular pathways both in vegetative and reproductive development. GO enrichment analysis showed that the above *OsSET* genes and their co-expressed genes seemed to particularly affect the same or similar GO categories. Promoter *cis*-elements identification and the combined analysis of expression correlation suggested that most of *OsSET* genes might be cell cycle regulated and were associated in the cell cycle progression by histone modifications via E2F**.** Moreover, we found that some MADS-box and APG proteins may be associated with OsSET on the regulation of cell differentiation and reproductive redevelopment in rice.

Although the studies of plant *SET* genes have received much progress, only a minority of *OsSET* genes has been verified in rice. The challenges still exist for the large number of genes in this family. It is a time-consuming process to molecular characterizes the functions and mechanisms of all *OsSET* genes in traditional approach. Thus our studies would provide valuable data for inferring the putative functions and pathways of the *OsSET* genes.

## Materials and Methods

### Identification of *OsSET* Members in Rice

Hidden Markov Model (HMM) profile of SET domain (PF00856) downloaded from Pfam (http://pfam.sanger.ac.uk/) were employed to identify the putative *OsSET* genes in rice (*Oryza. sativa*) [92]. The BlastP search was carried out using the HMM profile on website of MSU RGAP (http://rice.plantbiology.msu.edu/) and KOME (http://cdna01.dna.affrc.go.jp/cDNA/), followed by removal of redundant sequences from the two databases. Meanwhile, the keyword “SET” was also performed in these databases. Additionally, the Pfam and SMART database (http://smart.embl-heidelberg.de/smart/batch.pl ) were used to confirm and make classification of each predicted SET protein.

### Chromosomal Localization and Gene Duplication

*OsSET* genes were mapped on rice chromosomes according to their positions available in MSU RGAP. The distribution of *OsSET* genes was drawn by MapInspect (http://www.plantbreeding.wur.nl/UK/software_mapinspect.html ). The duplicated genes were elucidated from the segmental genome duplication of rice (http://rice.plantbiology.msu.edu/segmental dup/500 kb/segdup 500 kb.shtml ), with the maximal length distance permitted between collinear gene pairs of 500 kb [93]. Tandem duplicates were defined as genes separated by five or fewer genes. The distances between these genes on the chromosomes were calculated and the percentage of sequence similarities between the proteins encoded by these genes were determined by MegAlign software 4.0 (MEGA4) [94].

### Phylogenetic Analysis of *OsSET* Family

The protein sequences of *OsSET* family and *Arabidopsis* SET domain group (SDG) were aligned using ClustalX (version 2.0) program. An un-rooted neighbor-joining [93] phylogenetic tree was constructed in ClustalX based on the full sequences of the proteins with default parameters from rice and *Arabidopsis*. Bootstrap analysis was performed using 1,000 replicates. The phylogenetic tree thus obtained was viewed using MEGA 4 software.

### Structural and Sequence Analysis of *OsSET* Genes

Information in gene structures, transcripts, full-length cDNA, BAC accessions for each gene and characteristics of corresponding proteins were procured from MSU RGAP, KOME and GRAMENE. Protein sequences of putative OsSET members collected from the MSU RGAP and KOME were analyzed by EXPASY PROTOPARAM tool (http://www.expasy.org/tools/protparam.html ). Information in the number of amino acids, molecular weight, theoretical isoelectric point (pI), amino acid composition, and instability index (instability index of >40 was considered as unstable) were obtained [95]. The conserved domains of the OsSET protein in rice were determined by PFam program.

Protein sequences were analyzed in the MEME program (http://meme.sdsc.edu/meme/cgi-bin/meme.cgi ) to confirm the conserved motifs. The MEME program was employed using the following parameters: number of repetitions-any, maximum number of motifs-200, optimum motif width set to >2 and <200.

Promoter sequences (−2000 bps) of *OsSET* family genes were obtained from the Rice Annotation Project (RAP) database (http://rapdb.dna.affrc.go.jp/tools/dump ). The *cis*-elements of promoters were identified using the PLACE Web Signal ScanPLACE (http://www.dna.affrc.go.jp/PLACE/signalup.html ) [96], [97].

### Genome-wide Expression analysis of *OsSET* Family

Expression profile data of *OsSET* gene family in 24 tissues for Minghui 63 were extracted from CREP database (http://crep.ncpgr.cn, Microarray data sets: GSE19024) [98]. Expression values of each gene were logarithm in Microsoft excel 2007 and cluster analyses were performed using J-express 2011 with euclidean distances and hierarchical cluster method of “complete linkage”. The average signal value of biological replicates for each sample was used for analysis. When more than one probeset was available for one gene, the higher signal value of the probesets was used for analysis. Expression level in each of the tissues was compared against the expression in seed using a student-*t* test. The genes up- or down-regulated by more than two-fold and with p values <0.05 were considered to be differentially expressed. The average expression of more than two biological replicates for each sample was used for analysis.

### Identification of Correlated Genes and Network Construction

The co-expression data were downloaded from the CREP database. The standard deviations for the expression level of each *OsSET* gene in 24 tissues were calculated. First, we ranked the genes according to the correlation coefficients and screen ones that were greater than 0.75 positively correlated with *OsSET* gene expression. Then the Pearson correlation coefficient (PCC) and the student-*t* test of candidate genes that we interested in were recalculated with R project (version 2. 14.1). As the permutation test done by Ouyang *et al*., PCC ≥0.7 were significant (α = 0.05), We mapped the correlated genes (at a more strict level, PCC ≥0.75, p value ≤0.05) to the network with Cytoscape v2.8.1 [69], [99]. GO enrichment was performed by Singular Enrichment Analysis (SEA) tool in agriGO (http://bioinfo.cau.edu.cn/agriGO/index.php ) with default parameters using the rice MSU6.1 genome annotation as background [100]. Statistical significance was determined using Fisher’s exact test and Yekutieli multi-test adjustment.

### Real-time PCR Analysis of Representative Genes in *OsSET* Family

Primers designed for the RT-PCR analysis were listed in Table S6. Samples were ground in liquid nitrogen using a mortar and pestle. Total RNA (4 µg) was isolated using a RNAiso (Takara) and treated with RNase-free DNase I (Takara) for 15 min to eliminate possible contaminating DNA. First strand cDNA was then reverse transcribed from total RNA with an oligo(dT)18 primer in a 20 µl reaction (diluted to 40 µl before use) using an M-MLV Reverse Transcriptase (Promega) according to the manufacturer’s instructions. Real-time quantitative PCR was carried out on ABI StepOneTM Real-time PCR instrument (Applied Biosystems), containing 5 µl of 2× SYBR Premix EX Taq (Takara), 0.5 µl of Rox Reference Dye II (Takara), 0.5 µl of the cDNA sample, 2 µM of each gene-specific primer, in a final volume of 10 µl. The reactions were carried out according to the following temperature profile: 95°C for 30 seconds, 40 cycles of 95°C for 5 seconds, and 60°C for 34 seconds.

### Plant Materials and Growth Conditions

A *japonica* rice variety of Zhonghua11 was used in this study. Plants were grown at long day under natural light.

## Supporting Information

Table S1 A list of 43 *OsSET* genes identified in rice and their sequences and protein characteristics.
(XLS)

Table S2 *OsSET* genes that localized on duplicated segments of the rice genome.
(XLS)

Table S3 *Cis*-elements analysis of *OsSET* gene promoters.
(XLS)

Table S4 Average signal values in 24 samples of 40 *OsSET* genes in Minghui 63.
(XLS)

Table S5 Table S5a. Results of differential expression analysis using seed as reference (Minghui63); Table S5b. Results of differential expression analysis in 7 d-old seedlings subjected to three phytohormone (NAA, GA3 and KT) treatments and plumule or radicle with light/dark regulation in Minghui63. Differential expression genes have been taken p value less than 0.05 and fold change >2 or <0.5. When fold change >2, regulation is up, and when fold change <0.5, regulation is down.
(XLS)

Table S6 A list of co-expression genes mentioned in network construction with p value less than 0.05.
(XLS)

Table S7 Primers used in the research.
(XLS)
